# Psychiatric history and breast mammography experience

**DOI:** 10.1192/j.eurpsy.2026.11545

**Published:** 2026-07-31

**Authors:** A. Samet, I. Chaari, W. Abid, Y. Fourti, F. Charfeddine, L. Aribi, N. Messedi, J. Aloulou

**Affiliations:** Psychiatric department “B”, Hedi Chaker University Hospital, Sfax, Tunisia

## Abstract

**Introduction:**

Psychiatric history may influence tolerance of medical procedures such as mammography.

**Objectives:**

To evaluate differences in mammography experience, specifically pain intensity and satisfaction with results communication, between women with and without a psychiatric history.

**Methods:**

We conducted an online survey of the general female population in Tunisia via a sponsored Facebook campaign (December 2023–February 2024). Psychiatric history was defined as prior psychiatric consultation. Pain (1–10) and satisfaction with results communication were assessed.

**Results:**

A total of 278 women participated. Participants had a mean age of 46 years (SD 8.9, range 20–84). Most were married (84.9%), university educated (78.4%), and of middle socio-economic status (66.5%). A total of 22.7% reported having consulted a psychiatrist at least once, most frequently for depression, anxiety, or stress-related problems. 4.3% were under current psychotropic medication. The mean pain score during mammography was 5/10 (SD 2.8), with 34% reporting severe pain (≥7/10). Women with psychiatric history reported higher pain (6.3 vs 4.8, p = 0.02) and were less likely to be satisfied with communication (48% vs 71%, p = 0.02). Regression confirmed psychiatric history as an independent predictor of higher pain (β = +1.1, p = 0.03).

**Conclusions:**

Among Tunisian women, a history of psychiatric disorders was associated with a less favorable mammography experience, with higher pain perception and lower satisfaction with results communication. Incorporating mental health support into screening programs may improve comfort and adherence.

**Disclosure of Interest:**

None Declared

